# PHYD prevents secondary dormancy establishment of seeds exposed to high temperature and is associated with lower PIL5 accumulation

**DOI:** 10.1093/jxb/ery140

**Published:** 2018-04-10

**Authors:** Catherine Martel, Logan K Blair, Kathleen Donohue

**Affiliations:** Department of Biology, Duke University, Durham, NC, USA

**Keywords:** *Arabidopsis thaliana*, germination, phenology, phytochrome, secondary dormancy, temperature, thermoinhibition

## Abstract

Dormancy cycling controls the seasonal conditions under which seeds germinate, and these conditions strongly influence growth and survival of plants. Several endogenous and environmental signals affect the dormancy status of seeds. Factors such as time, light, and temperature influence the balance between abscisic acid (ABA) and gibberellic acid (GA), two phytohormones that play a key role in seed dormancy and germination. High temperatures have been shown to increase ABA level and prevent seed germination, a process known as thermoinhibition. High temperature can also cause the acquisition of secondary dormancy, preventing germination of seeds upon their return to favorable germination conditions. The mechanisms and conditions linking thermoinhibition and secondary dormancy remain unclear. Phytochromes are photoreceptors known to promote seed germination of many plant species including *Arabidopsis thaliana*. Here, we demonstrate a role for *PHYD* in modulating secondary dormancy acquisition in seeds exposed to high temperature. We found that a functional *PHYD* gene is required for the germination of seeds that experienced high temperature, and that ABA- and GA-related gene expression during and after pre-incubation at high temperatures was altered in a *phyD* mutant. We further show that the level of *PHYD* mRNA increased in seeds pre-incubated at high temperature and that this increase correlates with efficient removal of the germination repressor PIL5.

## Teaser

Acquisition of secondary dormancy enables spatio-temporal control of germination. Evidence suggests that PHYD influences secondary dormancy induction by high temperature in Arabidopsis and correlates with removal of the germination repressor PIL5.

## Introduction

Phenology, the timing of a plant’s life events such as germination and flowering, has a major impact on survival and fitness (Chuine and Beaubien, 2001; Walther *et al.*, 2002; Parmesan, 2006; Bradshaw and Holzapfel, 2008; Donohue *et al.*, 2010). Adaptive phenology ensures that each stage occurs during a season in which it can survive and progress to the next stage. For example, *Arabidopsis thaliana* (Arabidopsis) seeds can survive under hot, dry conditions, whereas the vegetative state cannot; rosettes are resistant to cold temperatures, whereas the reproductive stage is susceptible to freezing damage. To accommodate these vulnerabilities, seeds of winter annuals are dispersed in the spring but only germinate in the autumn, thus allowing seedlings to avoid summer drought. Similarly, flowering occurs after winter in the following spring, allowing reproductive buds to avoid freezing temperatures. These delays allow each life stage to avoid the seasonal conditions that could cause its mortality. Germinating at the appropriate time of year is the first crucial developmental transition that determines survival, and it is under strong natural selection in numerous plant species including Arabidopsis (reviewed in Donohue *et al.*, 2010).

The timing of seed germination depends on the dynamics of dormancy induction and breakage, both of which are influenced by the environment. Dormancy is described as the absence of germination under conditions that would otherwise permit germination. It evolved to prevent germination under temporarily adequate but seasonably inappropriate conditions, such as a cool, wet day in an otherwise hot and dry summer (Baskin and Baskin, 1998; Fenner and Thompson, 2005; Finch-Savage and Leubner-Metzger, 2006). Primary dormancy is established during seed development and maturation (Hilhorst, 1995), and secondary dormancy refers to the reacquisition of dormancy in dispersed seeds that have lost their primary dormancy (reviewed in Bewley, 1997; Donohue, 2009). Dispersed seeds cycle between different depths of dormancy in response to several factors, including time (after-ripening) and environmental cues such as temperature, light, and nitrate concentration (Bewley *et al.*, 2013; Footitt *et al.*, 2013). Dormancy status influences the conditions under which germination can proceed, with less dormant seeds being able to germinate under a broader range of environmental conditions (Baskin and Baskin, 1998). In this manner, dormancy dynamics can precisely influence the timing and seasonal conditions of germination.

Two phytohormones—gibberellic acid (GA) and abscisic acid (ABA)—are the major regulators of dormancy. GA stimulates growth of the embryo by positively regulating processes such as cell division and elongation, mobilization of carbon and energy reserves, and weakening of seed barriers to facilitate radicle emergence (Ogawa *et al.*, 2003; Finkelstein *et al.*, 2008). In contrast, ABA inhibits GA action and is associated with the imposition and maintenance of primary and secondary dormancy (Yoshioka *et al.*, 1998; Nambara and Marion-Poll, 2003; Ali-Rachedi *et al.*, 2004; Gulden *et al.*, 2004; Lin *et al.*, 2007; Toh *et al.*, 2008). Biosynthetic and catabolic enzymes dictate ABA and GA levels in seeds in response to developmental and environmental signals (Koornneef *et al.*, 2002; Toh *et al.*, 2012).

Much progress has been made in elucidating the genetic basis of primary dormancy established during seed maturation (reviewed in Finch-Savage and Leubner-Metzger, 2006; Finkelstein *et al.*, 2008), yet dormancy cycling, notably the induction of secondary dormancy, is less understood despite its critical role in the timing of germination under seasonally variable conditions. Both high and low temperatures are important regulators of dormancy in the seed bank (Baskin and Baskin, 1998; Probert, 2000; Fenner and Thompson, 2005). High temperature has a direct and indirect effect on seed germination: thermoinhibiton and secondary dormancy induction, respectively. Thermoinhibition refers to the inability of non-dormant seeds to germinate at high temperature. Thermoinhibited seeds can resume their germination program immediately following a return to favorable temperatures (Vidaver and Hsiao, 1975; Corbineau *et al.*, 1988; Toh *et al.*, 2008; Chiu *et al.*, 2012). Secondary dormancy induction can be triggered by exposure to high temperature, but it is distinguished from thermoinhibition by the lack of germination following a return to favorable conditions. As such, exposure to thermoinhibitory temperature may or may not result in secondary dormancy induction. Conditions and molecular mechanisms linking thermoinhibition and secondary dormancy remain unclear. Oat (*Avena sativa*), barley (*Hordeum vulgare*), and *Chenopodium bonus-henricus L*. seeds that have been exposed to thermoinhibitory temperatures fail to germinate following the return of permissive temperatures (Khan and Karssen, 1980; Corbineau *et al.*, 1988; Leymarie *et al.*, 2008). In Arabidopsis, thermoinhibition occurs at temperatures >30 °C (Burghardt *et al.*, 2016), and those temperatures have been shown to induce dormancy in partially dormant and completely non-dormant seeds (Auge *et al.*, 2015). Thermoinhibition results from an increase in ABA level and is mediated by increased expression of ABA-related genes such as the transcription factor *ABI5*, the germination repressor *SOMNUS* (*SOM*) (Lim *et al.*, 2013), and the ABA biosynthetic gene *9-cis-EPOXYCAROTENOID DIOXYGENASE9* (*NCED*) (Toh *et al.*, 2008; Chiu *et al.*, 2012; Lim *et al.*, 2013).

In Arabidopsis and other species, non-dormant seeds require light and favorable temperature to initiate germination. Phytochromes are photoreceptors that sense red and far-red light, and are involved in numerous developmental processes, from germination to flowering (Schafer and Bowle, 2002; Casal *et al.*, 2003). In non-dormant seeds, exposure to red light activates phytochromes, which degrade the basic helix–loop–helix (bHLH) repressor PIL5, thus promoting GA accumulation (increased biosynthesis and decreased catabolism) (Oh *et al.*, 2006), and ABA reduction (Shinomura *et al.*, 1994; Yamaguchi, 1998; Oh *et al.*, 2004). In Arabidopsis, the phytochrome family is composed of five genes, *PHYA*–*PHYE* (Clack *et al.*, 1994; Mathews and Sharrock, 1997), having both specific and overlapping activities. Each phytochrome has a different function in seed germination, with regard to responses to light quality (Ballaré *et al.*, 1990; Casal and Sánchez, 2008) and temperature (Heschel *et al.*, 2007, 2008; Donohue *et al.*, 2008). Diversification of the function of individual members of a gene family can contribute to the precise environmental regulation of developmental processes. Elucidating the genetic pathways whereby specific members of gene families act on developmental responses to specific environmental cues contributes to an understanding of environmentally modulated development.

Here, we investigate the genetic pathways involved in secondary dormancy induction by high temperature and the contributions of individual members of the phytochrome gene family. Specifically, we aim to: (i) characterize the temperature dependence of secondary dormancy induction, and test whether disruption of different phytochromes alters secondary dormancy induction by high temperature; (ii) test the involvement of phytochromes in this response by measuring the expression of the different phytochromes at different temperatures of seed pre-incubation; (iii) determine the contributions of GA and ABA hormonal pathways to this response by measuring the expression of GA and ABA biosynthetic and catabolic genes at different temperatures of seed pre-incubation; and (iv) test whether the specific phytochrome identified in aims (i) and (ii), namely PHYD, influences the expression of dormancy-related genes, by measuring gene expression and the abundance of PIL5 protein in the wild-type and a *phyD* mutant.

## Materials and methods

### Plant growth conditions

Genotypes and sources of wild-type (wt) and *phy* mutant seeds were previously described (Donohue *et al.*, 2008). All mutants were derived from the Landsberg *erecta* (L*er* hereafter) background. *phyA* is *phyA-1*, *phyB* is *phyB-1*, *phyD* is *phyD-1* (Aukerman *et al.*, 1997), and *phyE* is *phyE-1*. The *phyC* mutant was not included in this study as it was not available to us, and *PHYC* was shown to be non-functional in the absence of other phytochromes (Hu *et al.*, 2013).

Arabidopsis seeds of each genotype were planted in Metromix 360 (Scotts Sierra, Marysville, OH, USA) and cold-stratified for 5 d to induce synchronous germination. Twelve replicates of each genotype were randomly distributed within a growth chamber (EGC Model GC8-2 Plant Growth Chambers, Chagrin Falls, OH, USA) at 22 °C under long-day conditions (14 h light/10 h dark). When 70% of the seeds had matured, water was withheld for 2 weeks and seeds of all genotypes were harvested simultaneously. Seeds were collected, allowed to dry, and stored for 3 months (after-ripening) at ambient temperature before germination assays.

### Germination assays

The first germination assay characterized temperature-dependent secondary dormancy induction in wt seeds. Wt L*er* seeds that had after-ripened for 3 months were pre-incubated in the dark in the presence of water for 4 d at one of four temperatures: 4, 22, 32, or 34 °C. After pre-incubation in the dark, seeds were transferred to 22 °C in a 12 h photoperiod to assess germination.

The second germination assay tested wt and, *phyA*, *phyB*, *phyD*, and *phyE* mutants. Seeds were pre-incubated in the dark at 4, 22, or 32 °C before being transferred to a 12 h photoperiod at 22 °C to assess germination.

Germination assays were performed by placing 15 seeds in a Petri dish containing 0.5% agar. Each genotype×temperature assay used nine Petri dishes (three biological replicates×three technical replicates). Each Petri dish was sealed with parafilm, wrapped in aluminum paper, and incubated at the specified pre-incubation temperature in a germination chamber (Percival Scientific Inc., Perry, IA, USA) for 4 d. After the dark pre-incubation period, Petri dishes were unwrapped and transferred to a 22 °C chamber with a 12 h photoperiod. Seeds were examined once per day to assess radicle protrusion for up to 10 d, after which germination had plateaued. Seed viability was determined at the end of each experiment by assessing firmness to touch (Baskin and Baskin, 1998). The final germination percentage (number of germinants/number of viable seeds) of each Petri plate was used for all analyses.

### Gene expression analysis

Seeds from three groups of 8–10 individual plants each were pooled into three biological replicates and after-ripened for 3 months. For each quantitative reverse transcription–PCR (qRT–PCR) time point analyzed, 25–50 mg of seeds of each replicate pool were deposited on a wet filter paper in 60 mm Petri dishes and incubated as described for the germination assays. Dry and pre-incubated seeds were collected at specific time points after the initiation of imbibition and flash-frozen in liquid nitrogen for RNA extraction. Seeds undergoing dark pre-incubation were collected in a dark room under green light. Frozen seeds were ground using a mortar and pestle in liquid nitrogen, and RNA was extracted following previously described protocols (Oñate-Sánchez and Vicente-Carbajosa, 2008), and further cleaned using a Qiagen RNeasy column following the manufacturer’s instructions (QIAGEN, CA, USA). qRT–PCR was performed using Applied Biosystems 1-step SYBR Green and the primers listed in Table 1. The transcript level was normalized to the *TIP41-like* gene (*AT4G34270*), whose expression was shown to remain stable throughout seed germination (Czechowski *et al.*, 2005; Footitt *et al.*, 2011).

**Table 1.** Sequences of primers used for qRT–PCR analysis

Name	TAIR	Orientation	Sequence (5'→3')
*ABI5*	AT2G36270	F R	GGGAAGGAAAAGAGTAGTGGAT CCACTGTATATGCTTGTTTTCTT
*TIP41-like*	AT4G34270	F R	GTGAAAACTGTTGGAGAGAAGCAA TCAACTGGATACCCTTTCGCA
*GA2ox2*	AT1G30040	F R	AATAACACGGCGGGTCTTCAAATCT TCCTCGATCTCCTTGTATCGGCTAA
*GA3ox1*	AT1G15550	F R	CGACCACCCGGACGCGACTA TGCGTTGGACAGGTAGCCCG
*GA3ox2*	AT1G80340	F R	ATTGGCTCTCCCCTCCACGATTT CCCGGCCCATTGTATGTCCTTTTC
*NCED6*	AT3G24220	F R	TGATCTTCCTTACCAAGTGAAGA CTTAGGATGCGCTATCACTGAA
*NCED9*	AT1G78390	F R	TAATGGTGTTCGTTCACGAC CTAACACAAAGCTTGCTTCG
*PHYA*	AT1G09570	F R	ACGCATTAGAAGGAACTGAGGAG GTAAGATCATGGGCTACAAAACACA
*PHYB*	AT2G18790	F R	GCAGTCCAACGGAGGCA CTTTAGCACAAATGAACCGTCTTC
*PHYD*	AT4G16250	F R	AGAGGAGGCAGGAGAGTGAA AATGAACCGTCATCTATGCTTTTG
*PHYE*	AT4G18130	F R	AAGCATTGAGGAAGGCAAG CATTGAGAGGCAGAGTTTTGA
*PIL5*	AT2G20180	F R	TGAATCCCGTAGCGAGGAAACAA TTCCACATCCCATTGACATCATCTG
*RD29A*	AT5G52310	F R	AGTGATCGATGCACCAGGCGT CGGAAGACACGACAGGAAACACC
*SOMNUS*	AT1G03790	F R	AGCAATCAGCGTCTCCATCTCCAG TCAAGTCAAGAGATCATTGACCCATCC

### ABA-dependent transcript level

A 25–50 mg aliquot of L*er* wt seeds (3 months after-ripened) was placed on a filter paper imbibed with 0, 10, or 100 µM ABA (mixed isomers, Sigma-Aldrich, St. Louis, MO, USA). ABA was dissolved in ethanol to a concentration of 100 mM, and subsequent dilutions were performed in water. The control treatment contained 0.0001% ethanol, equivalent to the amount of ethanol present in the 10 µM ABA solution. Plates were sealed with parafilm and incubated at 22 °C under a 12 h photoperiod. Seeds were collected 24 h later and RNA was extracted as described above. Transcript levels of *PHYD* and *RD29a* were quantified by qRT–PCR as described above.

### Statistical analysis

To test for significant effects of pre-incubation temperature on germination rates and proportions, germination proportions at each time point were analyzed using logistic regression (PROC LOGISTIC in SAS 9.4; SAS Institute) using Fisher’s scoring optimization (ML) algorithm with Type-III likelihood ratio tests. The Firth’s penalized likelihood was used to accommodate issues of quasi-separation caused by extreme germination proportions (0 or 100%) in some treatments. The total number of germinants (successes)/the total number of viable seeds (trials) was the dependent variable for all analyses of germination proportions. To test whether phytochrome disruption altered germination responses to pre-incubation temperature, we tested for genotype×temperature interactions in logistic regression models of germination proportions, with genotype, temperature, and their interaction as factors; the wt was the reference genotype. To test whether pre-incubation temperature altered gene expression in wt seeds, normalized gene expression levels were analyzed with ANOVA, with time, temperature, and their interactions as fixed factors. To test whether ABA concentration altered gene expression of *PHYD* and *RD29a*, normalized gene expression was compared across ABA concentrations. To test whether wt and *phyD* genotypes differed in gene expression, normalized gene expression was compared between genotypes over time using ANOVA, with genotype, time, and their interaction included as fixed factors.

### Protein extraction and western blot

L*er* and *phyD* seeds were pre-incubated in the dark at 32 °C for 4 d as described in the germination assay, then transferred to permissive germination conditions (22 °C, 12 h photoperiod). Samples were collected at 0, 1, 2, 3, and 4 d following transfer to germination conditions. Seeds were ground using a mortar and pestle in liquid nitrogen. Proteins were extracted as described previously (Galvão *et al.*, 2012; Qiu *et al.*, 2015). Protein extracts were loaded on an SDS–polyacrylamide gel and transferred to a nitrocellulose membrane (Bio-Rad, CA, USA). Anti PIL5 (PIF1) polyclonal antibody (Qiu *et al.*, 2015) was used to detect PIL5 protein levels. PIL5 protein band intensities were quantified using ImageJ software.

## Results

### Phytochromes B and D are necessary to prevent high temperature-induced secondary dormancy

To examine the effect of pre-incubation temperatures on secondary dormancy induction, seeds that had after-ripened for 3 months were pre-incubated in the dark at different temperatures, then transferred to permissive germination conditions (22 °C with a 12 h photoperiod) and monitored for germination by measuring radicle emergence. L*er* wt seeds that had after-ripened for 3 months were used throughout these experiments since they have little residual primary dormancy compared with fresh seeds (see Supplementary Fig. S1 at *JXB* online).

**Fig. 1.**
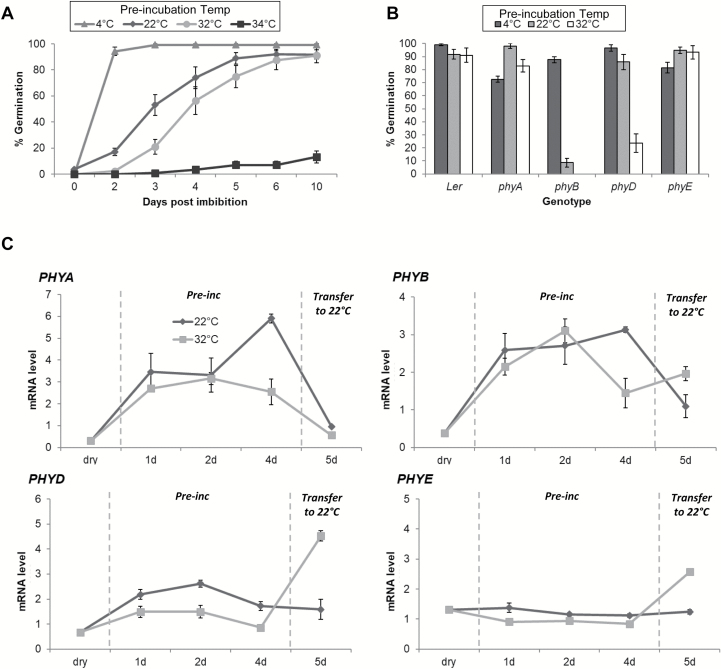
Role of phytochromes in germination at different temperatures. (A) Effect of temperature on germination. L*er* wt seeds that had after-ripened for 3 months were pre-incubated on agar in the dark for 4 d at the indicated temperature (4, 22, 32, or 34 °C). After 4 d, seeds were transferred to 22 °C with a 12 h photoperiod, and radicle protrusion was monitored daily. Error bars represent the SE of nine replicates of 15 seeds each. The speed of germination depended on temperature (germination×temperature interaction: Wald χ^2^=36.46, *P*=0.006, df=18). (B) Effect of phytochrome mutants on germination. L*er* wt seeds and phytochrome mutant seeds (after-ripened for 3 months) were pre-incubated on agar in the dark for 4 d at the indicated temperature (4, 22, or 32 °C). After 4 d, seeds were transferred to 22 °C and a 12 h photoperiod, and radicle protrusion was monitored after 10 d. Error bars represent the SE of nine replicates of 15 seeds each. Differences among genotypes depended on temperature (genotype×temperature interaction: Wald χ^2^=76.41, *P*<0.001, df=8; see Supplementary Table S1 for comparisons between L*er* wt and mutant genotypes at each temperature). (C) Effect of temperature on *PHY* gene expression. Relative expression level of *PHY* genes in L*er* wt seeds at different time points: dry seeds, 96 h pre-incubation (Pre-inc), and 24 h after transfer to permissive germination conditions (22 °C, 12 h photoperiod). mRNA levels were normalized to the *AT4G34270* (*TIP41-like*) transcript. Error bars indicate the SE of three biological replicates. See Supplementary Table S2 for analysis of differences in transcript levels between temperature treatments.

L*er* seeds eventually germinated to almost 100% following pre-incubation at 4, 22, and 32 °C, but only to 10% following pre-incubation at 34 °C (Fig. 1A). This indicates that pre-incubation at 34 °C induced secondary dormancy in L*er* wt seeds. A closer examination of the germination time course reveals that seeds pre-incubated at 4 °C germinated the fastest (within 2 d of light treatment), whereas seeds pre-incubated at 32 °C showed a 1 d delay in germination compared with seeds pre-incubated at 22 °C (Fig. 1A).

We next compared the germination ability of seeds from different phytochrome mutants after dark pre-incubation at 4, 22, and 32 °C and subsequent transfer to 22 °C in the light. As expected from previous studies (Heschel *et al.*, 2007; Donohue *et al.*, 2008), *phyB* mutant seeds failed to germinate in all treatments except 4 °C (Fig. 1B; Supplementary Table S1). The *phyA* and *phyE* mutants behaved like wt L*er* seeds under the conditions examined. Interestingly, the *phyD* mutant showed a <25% germination proportion after a 32 °C pre-incubation compared with >90% germination for wt seeds under the same conditions. Therefore, *PHYB* is necessary for germination across many temperatures, whereas *PHYD* is required for germination specifically after a 32 °C pre-incubation treatment.

### Pre-incubation in the dark at 32 °C triggers PHYD expression

To test whether the contribution of *PHYD* to germination after pre-incubation at 32 °C in the dark could be mediated by an increase in *PHYD* gene expression, we compared *PHYD* mRNA transcript levels during and after pre-incubation at 22 °C and 32 °C. We also measured the transcript level of other phytochromes for comparison. Temperature had a small influence on *PHYD* transcript levels during dark pre-incubation, but a 4- to 5-fold up-regulation was observed following transfer to permissive germination conditions in seeds that had been pre-incubated at 32 °C (Fig. 1C; Supplementary Table S2). This increase in transcript level was not seen in seeds pre-incubated at 22 °C. Therefore, *PHYD* is specifically up-regulated after a hot, dark pre-incubation period upon transfer to permissive germination conditions (in the light). A second round of qRT–PCR focusing on later germination time points was performed to further characterize *PHYD* expression following hot pre-incubation. This assay confirmed that *PHYD* transcription increased following pre-incubation at 32 °C in the dark, peaking 24 h after transfer to permissive germination conditions (Supplementary Fig. S2). In contrast, *PHYA* and *PHYB* transcript levels increased during pre-incubation in the dark at both 22 °C and 32 °C, with seeds pre-incubated at 22 °C showing a >2-fold increase in transcript level in the dark compared with seeds pre-incubated at 32 °C (Fig. 1C; Supplementary Table S2). Interestingly, the *PHYE* transcript level also increased following hot dark pre-incubation, even though *PHYE* disruption did not alter germination after hot pre-incubation.

Combined, these results indicate that the transcription of phytochromes changes during dark pre-incubation at 32 °C. *PHYA* and *PHYB* are up-regulated in the dark at both temperatures, whereas *PHYD* expression is strongly up-regulated after pre-incubation at 32 °C and transfer to light and permissive temperature. Therefore, the involvement of *PHYD* in germination after hot pre-incubation appears to be, at least in part, influenced by its temperature-dependent expression.

### Dark pre-incubation at 32 °C increases mRNA transcript levels of ABA-related genes and decreases those of GA-related genes

**Fig. 2.**
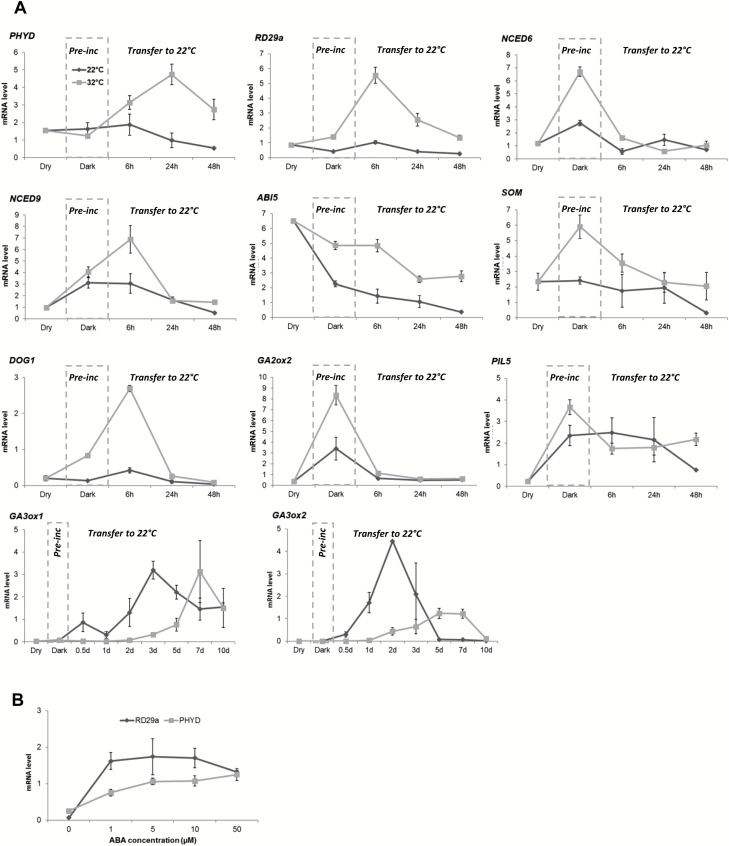
mRNA levels during and after pre-incubation in the dark at high temperature. (A) Effect of pre-incubation at 32 °C in the dark on mRNA levels in L*er* wt seeds. The relative mRNA level was measured in dry seeds, 96 h after dark pre-incubation (Pre-inc) at 22 °C or 32 °C, and at 6, 24, and 48 h following transfer to permissive germination conditions at 22 °C and a 12 h photoperiod. mRNA levels of the focal genes were normalized to transcript levels of the reference gene *TIP41-like* (*AT4G34270*). For *GA3ox1* and *GA3ox2*, time points (0.5d–10d) correspond to days after transfer to permissive germination conditions. See Supplementary Table S3 for analysis of differences in mRNA levels between temperature treatments. (B) The *PHYD* mRNA level is regulated by ABA. *PHYD* and *RD29a* mRNA levels in L*er* wt seeds incubated at 22 °C for 24 h (12 h photoperiod) in the presence of 0, 1, 5, 10, and 50 µM ABA. mRNA levels were normalized to the reference gene, *TIP41-like* (*AT4G34270*) transcript. Error bars indicate the SE of three biological replicates. Test for effect of ABA concentration: *PHYD F*-value=11.95, *P*<0.001, df=4; *RD29a F*-value=7.47, *P*=0.005, df=4.

To identify the major hormonal pathways involved in germination responses to pre-incubation at high temperature, we used qRT–PCR to examine the transcript level of representative germination- and dormancy-associated genes within the ABA and GA pathways in wt seeds. For subsequent analysis, we compared transcript levels in seeds that were pre-incubated in the dark at 22 °C versus 32 °C and then transferred to permissive germination conditions for up to 4 d. As described above, *PHYD* expression increased upon transfer to light and 22 °C if seeds were pre-incubated in the dark at 32 °C, but it remained low in seeds pre-incubated in the dark at 22 °C (Figs 1C, 2A; Supplementary Table S3). ANOVAs revealed significant effects of temperature, time, and time×temperature interaction on transcript levels of most genes (Supplementary Table S3).

Because thermoinhibitory temperatures have been shown to induce a strong increase in ABA levels (Toh *et al.*, 2008), we tested whether dark pre-incubation at 32 °C triggered an increase in ABA levels that persisted after transfer to permissive conditions, by measuring the transcript levels of the ABA reporter gene *RD29a* (Nakashima *et al.*, 2006). As shown in Fig. 2A, we observed a >5-fold increase in *RD29a* transcript level following a 32 °C dark pre-incubation, consistent with an increase in ABA levels. Up-regulation of the ABA biosynthetic genes *NCED6* and *NCED9* could contribute to the increase in ABA (Fig. 2A). *NCED6* showed increased expression during the dark pre-incubation phase and returned to the basal level after transfer to permissive germination conditions. This pattern contrasted with that of *NCED9*, whose expression increased only after transfer to permissive germination conditions following a period of pre-incubation at 32 °C. Nonetheless, both ABA biosynthesis genes were up-regulated by pre-incubation at 32 °C, consistent with the higher transcript level of *RD29a* at that pre-incubation temperature.

*ABI5* encodes a transcription factor that mediates the effects of ABA. Its expression was high in dry seeds and gradually declined during pre-incubation and germination at both temperatures tested, but the decline was more pronounced in seeds pre-incubated at 22 °C compared with 32 °C, suggesting that ABA responsiveness would be higher in seeds pre-incubated at 32 °C. *SOMNUS* (*SOM*) is a key repressor of seed germination in the dark and has recently been shown to be up-regulated in thermoinhibited seeds by ABI3, ABI5, and the DELLA transcription complex (Lim *et al.*, 2013). *SOM* gene expression was up-regulated in seeds pre-incubated at 32 °C in the dark. *SOM* then returned to basal levels following transfer to permissive germination conditions in seeds pre-incubated at both 22 °C and 32 °C.

Expression of *DOG1* correlates with both primary (Teng *et al.*, 2008; Chiang *et al.*, 2011; Mortensen *et al.*, 2011; Mortensen and Grasser, 2014) and secondary dormancy (Footitt *et al.*, 2011, 2013). Recently, *DOG1* was shown to be involved in an ABA-independent thermoinhibition (Huo *et al.*, 2016). *DOG1* expression was briefly up-regulated >2-fold following pre-incubation at 32 °C, peaking at 6 h after transfer to permissive germination conditions before returning to basal levels by 24 h.

The GA-catabolizing gene *GA2ox2* showed increased expression during dark pre-incubation at 32 °C compared with 22 °C, suggesting that pre-incubation at high temperature leads to decreased GA content via GA degradation. GA3ox1 and GA3ox2 enzymes promote the synthesis of bioactive GA and thereby promote seed germination (Yamaguchi, 1998; Mitchum *et al.*, 2006). Since seeds pre-incubated at 32 °C showed delayed germination (Fig. 1A), we extended our time course to 10 d after transfer to permissive germination conditions in order to capture full *GA3ox1/2* expression patterns (Fig. 2A). Transcript levels of *GA3ox1* increased around the seventh day after transfer to permissive germination conditions in seeds pre-incubated at 32 °C compared with the third day for seeds pre-incubated at 22 °C. A similar delay in *GA3ox2* transcript accumulation was also observed, with a higher expression between the fifth and seventh day in seeds pre-incubated at 32 °C compared with the second day in seed pre-incubated at 22 °C. The shift in *GA3ox1/2* expression levels reflects the delayed germination phenotype observed in seeds pre-incubated at 32 °C (Fig. 1A).

The major germination repressor *PIL5* was up-regulated upon pre-incubation at both 22 °C and 32 °C, and its expression remained similar between both seed populations for the first 24 h following transfer to permissive germination conditions. At 48 h, *PIL5* expression dropped in seeds pre-incubated at 22 °C but remained high in seeds pre-incubated at 32 °C, although this difference was not significant (Supplementary Table S3).

Taken together, these results suggest that pre-incubation at 32 °C enhanced ABA biosynthesis and GA degradation in the dark and led to delayed *GA3ox1* and *GA3ox2* expression upon transfer to permissive germination temperature. Thus, wt seeds pre-incubated at 32 °C seem to have acquired a weak secondary dormancy that was broken by transfer to light and permissive germination temperature.

### ABA concentration correlates with PHYD expression level

Comparison of gene expression patterns of seeds pre-incubated at 32 °C, described above, suggests that an increase in ABA levels could induce *PHYD* expression that could then oppose secondary dormancy induction in wt seeds. To test this possibility, we measured *PHYD* transcript levels in the presence of different concentrations of ABA under permissive germination conditions (22 °C, 12 h photoperiod). As shown in Fig. 2B, the *RD29a* transcript level increased in the presence of ABA, indicating that exogenous ABA could penetrate the imbibing seeds and influence transcription. Interestingly, the *PHYD* transcript level correlated with ABA concentration, indicating that ABA can up-regulate *PHYD* expression independently of temperature.

### PHYD modulates PIL5 protein level and germination-associated gene expression

**Fig. 3.**
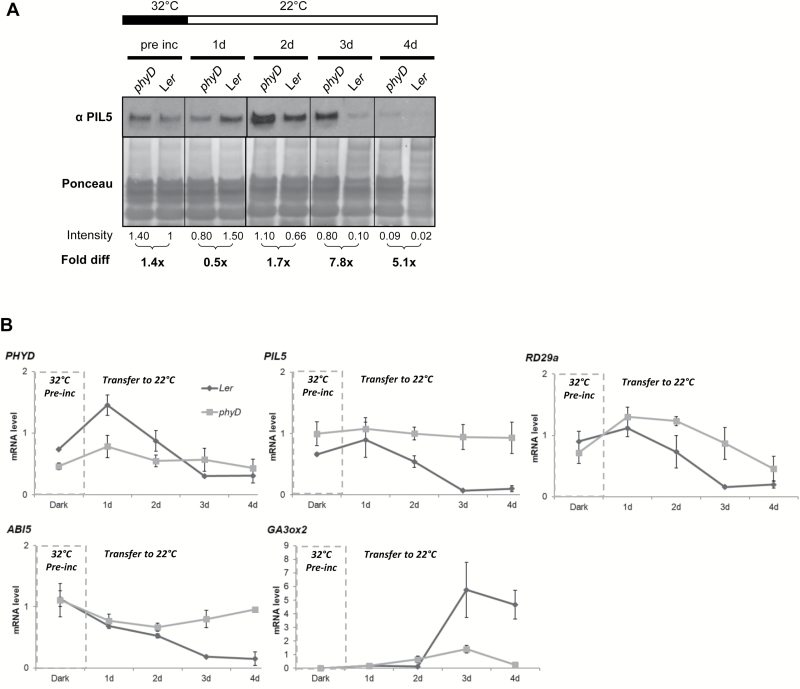
Effect of *phyD* mutation on PIL5 protein stability and mRNA levels after 32 °C pre-incubation. (A) *PHYD* influences PIL5 levels. PIL5 levels in L*er* and *phyD* mutant seeds during pre-incubation in the dark at 32 °C and 1, 2, 3, and 4 d after transfer to permissive germination conditions (22 °C, 12 h photoperiod). Intensity: relative intensity of PIL5 protein level compared with L*er* pre-incubated sample (measured by ImageJ software). Fold diff: fold difference in PIL5 protein levels between L*er* and *phyD* samples at each time point. (B) Effect of *phyD* mutation on mRNA levels. mRNA levels in L*er* wt and *phyD* mutant seeds during pre-incubation in the dark at 32 °C and 1, 2, 3, and 4 d after transfer to permissive germination conditions (22 °C, 12 h photoperiod). mRNA levels were normalized to the *TIP41-like* (*AT4G34270*) transcript. Error bars indicate the SE of three biological replicates. See Supplementary Table S4 for results of analysis of differences in gene expression between L*er* wt and *phyD* mutant genotypes.

PHYA and PHYB promote germination by interacting with and inducing the degradation of the germination repressor PIL5 (Oh *et al.*, 2004). To test if increased *PHYD* expression increased PIL5 degradation, we compared PIL5 protein levels in wt and *phyD* seeds pre-incubated in the dark at 32 °C. Since *PHYD* expression peaked 24 h after transfer to permissive germination conditions (Fig. 2A), we measured PIL5 protein levels during dark pre-incubation and after 1, 2, 3, and 4 d following transfer to permissive germination conditions. The PIL5 protein level was more slowly degraded in *phyD* seeds compared with wt seeds (Fig. 3A). PIL5 protein was still detected up to 3 d after transfer to permissive germination conditions in the *phyD* mutant, whereas it was barely detectable in wt seeds at a corresponding time point. This decrease in PIL5 protein in wt seeds after 3 d in permissive conditions is consistent with a pronounced increase in germination proportion of wt seeds at that time (Fig. 1A). Thus, functional *PHYD* appears to promote the degradation of PIL5 after pre-incubation at 32 °C.

To test whether *PHYD* influences the expression of germination-associated genes when seeds experienced pre-incubation at 32 °C, we examined the expression level of *PHYD*, *PIL5*, and a subset of downstream targets of PIL5 in L*er* wt and *phyD* mutant seeds using qRT–PCR. *PHYD* expression was lower in the *phyD* mutant, as expected (Fig. 3B; Supplementary Table S4) (Aukerman *et al.*, 1997). Interest ingly, the *PIL5* transcript level remained higher in the *phyD* mutant compared with wt seeds after transfer to permissive germination conditions, suggesting that *PHYD* may directly or indirectly repress *PIL5* expression. The expression of *ABI5*, a direct target of PIL5 (Oh *et al.*, 2009), decreased over time in L*er* wt seeds, but remained high in *phyD* mutant seeds, consistent with higher levels of *PIL5* gene expression and higher levels of PIL5 protein in *phyD* seeds. ABA levels or sensitivity to ABA, as detected by the reporter gene *RD29a*, decreased more rapidly in wt than in *phyD* mutant seeds, also consistent with higher levels of PIL5 protein in *phyD* seeds. *GA3ox2* expression increased at 3 d in wt seeds but remained very low in *phyD* seeds, consistent with the absence of germination in those seeds. *GA3ox1* remained low in both wt and *phyD* seeds at the time points examined (Supplementary Fig. S3).

Taken together, these results suggest that increased *PHYD* expression influences ABA and GA responses by influencing PIL5 protein levels in seeds pre-incubated at high temperature, following their return to favorable germination conditions.

## Discussion

Thermoinhibition is a process that occurs at high temperature to prevent germination of seeds under unfavorable temperatures. Secondary dormancy, in contrast, correspond to a lack of germination under favorable conditions. We show that exposure to high temperature triggered the establishment of secondary dormancy, impeding seed germination even after a return to permissive germination conditions. The depth of secondary dormancy in wt seeds was proportional to the temperature experienced. Pre-incubation at 32 °C appeared to induce a shallow dormancy that was overcome by permissive germination conditions, but pre-incubation at 34 °C induced a deeper dormancy that was not removed by transfer to permissive conditions. We showed that the ability to overcome shallow 32 °C-induced dormancy required a functional *PHYD* gene, as seeds with non-functional *PHYD* remained in a dormant state even after transfer to permissive conditions. *PHYD* reduced the amount of the germination repressor PIL5 protein following transfer to permissive conditions. When *PHYD* was non-functional (in *phyD* mutant seeds), PIL5 protein persisted in seeds pre-incubated at 32 °C following transfer to permissive germination conditions, and such seeds maintained higher ABA biosynthesis and decreased GA synthesis, leading to lower germination. Therefore, *PHYD* influences the upper temperature seeds can experience without entering deep secondary dormancy.

### Effect of high temperature pre-incubation on gene expression

Previous studies have focused on thermoinhibition, in which seed germination at high temperature is prevented (Toh *et al.*, 2008; Lim *et al.*, 2013). To expand on these studies, we examined seeds transiently exposed to high temperatures, but subsequently returned to permissive germination conditions. This allowed the effect of high temperature on secondary dormancy induction to be studied. It is unclear how thermoinhibition and secondary dormancy processes are related and whether they are controlled by the same genes.

One may expect genes involved in thermoinhibition to be influenced by high temperature, but that their expression would return to previous levels once seeds are returned to favorable conditions. In contrast, genes involved in secondary dormancy induction and maintenance should remain or become differentially expressed even after seeds are returned to permissive germination conditions. We found that several genes previously associated with dormancy and thermoinhibition had altered gene expression in wt L*er* seeds after exposure to high temperatures. Because wt seeds did eventually germinate after pre-incubation at 32 °C, it is unclear whether the observed delay in germination of wt seeds represents weak secondary dormancy that was broken by permissive conditions, or whether it represents thermoinhibition that persisted even after transfer to permissive conditions. The observation that wt seeds were induced into persistent secondary dormancy by pre-incubation at only a slightly higher temperature (34 °C) suggests that shallow dormancy is a likely possibility, and the continuum between persistent effects of thermoinhibition and secondary dormancy itself suggests that these processes are closely related.

After seeds were pre-incubated at high temperature, we detected an up-regulation of the ABA biosynthetic gene *NCED6*, the ABA signaling gene *ABI5*, the GA-degrading gene *GA2ox2*, and the dormancy-related genes *SOM* and *DOG1*. Interestingly, *GA2ox2*, *NCED6*, and *SOM* expression returned to basal levels as soon as seeds were placed under permissive germination conditions, suggesting a role in thermoinhibition but not in maintaining secondary dormancy. In contrast, *ABI5* expression remained high during both the dark pre-incubation and germination phases in seeds pre-incubated at 32 °C, consistent with ABA’s role in controlling both thermoinhibition and secondary dormancy. Barley seeds induced into secondary dormancy by high temperatures were also shown to accumulate high levels of ABA both during high temperature (30 °C) exposure and following transfer to permissive germination conditions (20 °C) (Leymarie *et al.*, 2008).

*NCED6* and *NCED9* are members of a multigene family regulating the key step in ABA biosynthesis. Although both genes have been shown to be expressed in Arabidopsis seeds, only the expression of *NCED9* was shown to increase during thermoinhibition by Toh *et al.* (2008). Our results show that *NCED6* expression is also up-regulated in response to high temperature in the dark. Up-regulation of *NCED6* in dark-imbibed Cvi seeds had previously been correlated with increased dormancy (Cadman *et al.*, 2006; Seo *et al.*, 2006; Footitt *et al.*, 2011). This pattern suggests that *NCED6* is involved in dormancy cycling in the seed bank; that is, in buried seeds that have restricted light, whereas *NCED9* is involved in the regulation of seed ABA levels in the light.

PIL5 is an important negative regulator of germination. We found similar levels of *PIL5* transcript during dark pre-incubation and the first day after transfer to light, regardless of pre-incubation temperature, but a slightly (but not significantly) higher transcript at 48 h after transfer to permissive conditions. This observation, coupled with the observed accumulation of PIL5 protein in the *phyD* mutant (Fig. 3, and discussion below), suggests that PIL5 protein levels could be critical in bridging thermoinhibition and secondary dormancy acquisition.

Expression of the GA biosynthetic genes *GA3ox1* and *GA3ox2* is positively modulated by light and precedes radicle emergence (Yamaguchi, 1998). We found a delay in the expression of *GA3ox1* and *GA3ox2* in seeds pre-incubated at 32 °C compared with seeds pre-incubated at 22 °C following their transfer to light. This delay correlates with the delay in germination observed in those seeds (Fig. 1A) and suggests that 32 °C induces a shallow dormancy that is overcome upon transfer to permissive conditions, whereas pre-incubation at higher temperatures (34 °C) leads to induction of a deeper dormancy that cannot be broken by light and permissive temperature.

In sum, dormancy induction by high temperature involves both the up-regulation of ABA-mediated dormancy pathways and up-regulation of the major gene involved in GA catabolism, resulting in a delay in germination-promoting GA biosynthesis.

### Role of PHYD

We found that a functional *PHYD* allele influenced the ceiling temperature that seeds can experience before acquiring secondary dormancy. Indeed, *phyD* mutant seeds failed to germinate after exposure to 32 °C, whereas wt seeds failed to germinate only after exposure to 34 °C, indicating that *PHYD* either prevents the establishment of secondary dormancy at 32 °C or can overcome the shallow secondary dormancy induced by this treatment. *PHYD* transcript remained low during dark pre-incubation regardless of temperature, but increased almost 3-fold in seeds pre-incubated at 32 °C after transfer to permissive germination conditions. This suggests that *PHYD* contributes to the removal of the shallow secondary dormancy established during pre-incubation at 32 °C. Previous studies have also reported a correlation between *PHYD* expression and dormancy levels in Cvi seeds (Cadman *et al.*, 2006; Finch-Savage *et al.*, 2007; Footitt *et al.*, 2013).

The mechanism by which *PHYD* influences germination after pre-incubation at high temperature could be mediated by its influence on the germination repressor PIL5. PIL5 protein was degraded faster in L*er* wt than in *phyD* mutant seeds pre-incubated at 32 °C following transfer to permissive germination conditions. Since wt seeds germinated to higher proportions (Fig. 1A), they are expected to contain lower levels of PIL5 protein because of the germination process (Lee *et al.*, 2014), partially explaining the lower levels of PIL5 protein observed in wt seeds. However, quantification of the PIL5 protein level indicates that (i) a difference in PIL5 intensity is observed as early as day 2 when very few wt seeds had germinated; and (ii) the difference in PIL5 protein intensity at day 3 between the wt and *phyD* is six times higher than expected if the only difference were the percentage of non-germinated seeds. Indeed, only ~20% of wt seeds had germinated by day 3 (see Fig. 1A), which translates into a 1.25-fold increase in ungerminated seed compared with the wt. The difference in PIL5 protein intensity between *phyD* and wt samples at day 3, however, reveals a 7.8-fold increase in the former (i.e. more than six times that expected from germination alone). This suggests that although a portion of the decrease in PIL5 protein level can be attributed to seed germination *per se*, most of the difference is attributable to the genotypic difference between the seeds.

PHYA and PHYB proteins directly interact with and promote the degradation of PIL5 (Oh *et al.*, 2004; Shen *et al.*, 2008). *PHYD* is the closest homolog of *PHYB*, and its ability to promote PIL5 degradation alongside *PHYA* and *PHYB* has already been reported (Shen *et al.*, 2008). We suggest that pre-incubation at high temperature leads to increased expression of *PHYD*, which is then able to induce the degradation of PIL5 protein, most probably by promoting its phosphorylation and subsequent ubiquitination. It is also possible that the higher PIL5 protein levels observed in *phyD* mutant seeds is caused by altered *PIL5* gene expression, since *PIL5* was expressed to slightly higher levels in *phyD* mutant seeds (Fig. 3). Interestingly, PIL5 protein eventually decreased to very low levels in *phyD* mutant seeds, even though the seeds never germinated, indicating that lack of germination is not solely due to the persistence of PIL5 protein. This is consistent with the low expression of *PIL5* observed in deeply dormant seeds during winter time in the field (Footitt *et al.*, 2011, 2013) and suggests that PIL5 provides a temporary block on germination but is not necessary for maintenance of the secondary dormant state.

**Fig. 4.**
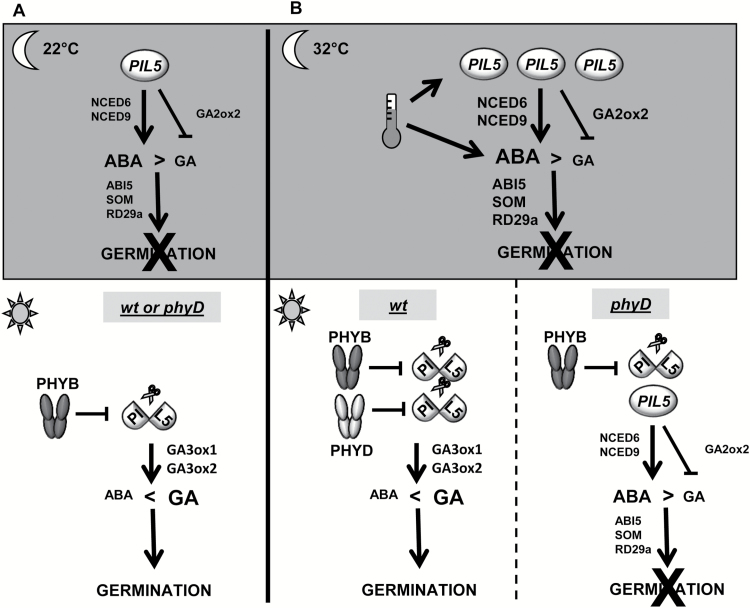
Germination model. (A) Dark pre-incubation at 22 °C leads to accumulation of PIL5 protein, which leads to higher ABA levels (via *NCED6* and *NCED9* expression) and responsiveness (via *ABI5* and *SOM*) and lower GA (via GA2ox2), which prevents germination. Upon transfer to light, PHYB degrades PIL5, allowing the accumulation of GA (via GA3ox1 and GA3ox2), which promotes germination. (B) Pre-incubation in the dark at high temperature (32 °C) leads to increased accumulation of PIL5 and/or PIL5 with higher stability, which strongly impedes germination via ABA accumulation and GA catabolism. Upon transfer to favorable germination conditions, PHYB and PHYD degrade PIL5, allowing GA accumulation and germination. PHYD might also repress the *PIL5* gene expression level. In the absence of PHYD (*phyD* mutant), PIL5 remains active and blocks germination.

We propose the following model to explain the role of *PHYD* in secondary dormancy induction by high temperature (Fig. 4). Under a permissive temperature (22 °C), seed pre-incubation leads to the accumulation of the germination repressor PIL5, a mechanism believed to prevent premature germination in the dark. Upon transfer to light, PIL5 is degraded by PHYB, thereby reducing ABA biosynthesis and ABA response, and impeding GA catabolism, ultimately allowing germination. Seed pre-incubation at 32 °C leads to higher and/or more stable PIL5 proteins that cannot be entirely degraded by *PHYB* under permissive conditions. Increased *PHYD* expression following 32 °C pre-incubation provides additional phytochrome activity to fully overcome PIL5-imposed germination repression by increasing PIL5 protein degradation and potentially through direct or indirect repression of *PIL5* gene expression, therefore allowing germination to proceed. PHYD could function as a homodimer or could heterodimerize with PHYB (Sharrock and Clack, 2004) to target PIL5, since *PHYB* is also necessary for germination following pre-incubation at 32 °C (Fig. 1B). In the absence of functional PHYD protein, the higher PIL5 protein level can delay germination until the establishment and/or consolidation of secondary dormancy. Whether PIL5 itself is necessary for secondary dormancy acquisition or maintenance of seeds pre-incubated at 32 °C or only allows a PIL5-independent pathway to complete secondary dormancy establishment remains to be tested. In addition, further study of interactions between *PHYD* and other genes involved in dormancy induction and maintenance would provide valuable information on the pathways whereby *PHYD* may contribute to the regulation of secondary dormancy. Additional studies of gene expression in *phyD* mutants and double mutant analysis would be especially informative.

### PHYD transcriptional regulation

We found that *ABI5* expression was higher in seeds pre-incubated at 32 °C than in those pre-incubated at 22 °C at time points preceding *PHYD* expression. The ABI3/ABI5/DELLA protein complex was implicated in gene regulation in response to high temperature, including the expression of the transcription factor gene *SOM* (Lim *et al.*, 2013). Although *PHYD* was not reported by Lim *et al.* (2013) as a putative target, it would be interesting to test whether the ABI3/ABI5/DELLA transcription complex is involved in its expression.

We found that ABA is directly implicated in the regulation of PHYD expression. We showed a positive correlation between ABA concentration and *PHYD* expression in seeds pre-incubated under favorable germination conditions, thus demonstrating that ABA can increase *PHYD* expression even in the absence of elevated temperature. This result is consistent with that of Okamoto *et al.* (2010), who reported an increase in *PHYD* and *PHYE* expression in seeds of genotypes that overproduced ABA.

*PHYD* expression increased only after transfer to permissive germination conditions in the light, suggesting that light is also needed for its up-regulation. A previous study suggests that light has little influence on phytochrome gene expression, including *PHYD* (Clack *et al.*, 1994). This study was performed on seedlings, and it remains possible that light regulation of phytochrome expression during seed germination was overlooked. In our experiments, the transfer to permissive germination conditions entailed altering both light (dark to light) and temperature (32 °C to 22 °C) simultaneously. Specifically, decoupling light from permissive germination temperature would permit a test of whether *PHYD* expression in seeds pre-incubated at high temperature requires light.

### Regulation of environment-dependent germination by duplicated genes

This study shows that phytochromes are involved in the ability to germinate after pre-incubation at high temperatures. *PHYD* is the most similar phytochrome gene to *PHYB* (Clack *et al.*, 1994; Mathews and Sharrock, 1997), yet it exhibits its own distinctive pattern of gene expression and temperature-dependent phenotypic effects. Whereas both *PHYB* and *PHYD* are required for germination after pre-incubation at 32 °C, only *PHYB* contributes to germination at other temperatures; *PHYD* disruption does not alter germination at lower temperatures (Heschel *et al.*, 2007, 2008; Donohue *et al.*, 2008). *PHYD* could be a major determinant of dormancy cycling dynamics in temperate climates in which hot summers are frequent, as it influences secondary dormancy induction by high temperatures. In this study, we showed that different phytochromes have distinctive patterns of gene expression in response to environmental cues, and that their contribution to the phenotype of germination depends on environmental conditions. We did not investigate the role of *PHYC* in high temperature germination as it was shown to be non-functional in the absence of other phytochromes (Clack *et al.*, 2009; Hu *et al.*, 2013). *PHYC* has since been shown to modulate *PHYA* activity during far-red-mediated germination (Arana *et al.*, 2014), and it would be interesting to test whether *PHYC* also influences germination regulation following high temperature exposure. Additional studies are necessary to compare the pathways whereby specific phytochromes may independently influence germination. This study, however, demonstrates that part of the diversification of germination regulation by specific phytochromes occurs at the level of gene expression.

In conclusion, we showed that *PHYD* is required to relieve the shallow dormancy imposed by exposure to 32 °C. Induction of secondary dormancy following exposure to high temperature shares a partial genetic basis with thermoinhibition, in that increased ABA biosynthesis, increased expression of an ABA response gene, and increased GA catabolism appear to be involved in both responses. Seeds’ ability to germinate after a return to permissive temperatures is associated with the degradation of PIL5 protein. *PHYD* has a distinctive pattern of gene expression in response to pre-incubation at high temperature and appears specifically to impede the maintenance of dormancy after hot pre-incubation. This highlights a functional diversification of phytochrome expression and function that can contribute to fine-tuned responses of germination to complex environmental scenarios.

## Supplementary data

Supplementary data are available at *JXB* online.

Table S1. Tests for differences between L*er* wild-type and mutant phytochrome genotypes at different temperatures.

Table S2. Tests for effect of temperature on transcript level over time.

Table S3. Tests for effect of temperature on transcript level during and after high temperature pre-incubation in the dark.

Table S4. Tests for differences between L*er* wild-type and the *phyD* mutant in transcript levels over time.

Fig. S1. Primary dormancy in fresh and 3 months after-ripened (AR) seeds of L*er* wild-type and *phyD* mutant genotypes.

Fig. S2. *PHYD* transcript level in L*er* wild-type seeds pre-incubated at two temperatures.

Fig. S3. *GA3ox1* transcript level following high temperature pre-incubation.

Supplementary Figures and Tables
