# Supplementary material for: PHYD prevents secondary dormancy establishment of seeds exposed to high temperature and is associated with lower PIL5 accumulation
Source: J Exp Bot. 2018 Apr 10;69(12):3157–69. doi: 10.1093/jxb/ery140 (PMC5972622; doi:10.1093/jxb/ery140)
Supplement: Supplementary Figures and Tables [file ery140_suppl_supplementary_figures_and_tables.pdf]

PHYD PREVENTS SECONDARY DORMANCY ACQUISITION

Catherine Martel, Logan K Blair, and Kathleen Donohue

SUPPLEMENTARY DATA

Supplementary Table S1: Tests for differences between L*er* wild-type and mutant phytochrome genotypes at different temperatures. Results of logistic regression of number of germinants/number of seeds. Differences between Ler wild-type (wt) and mutant phytochromes depended on temperature (Genotype x Temperature interaction: Wald Chi-square = 76.41, P < 0.001, df = 8). See Figure 1B for mean germination proportions. Wald Chi-square is shown comparisons between wt and each mutant separately. *P < 0.05; ** P < 0.01; ***P < 0.001. df = 1.

Contrast	4ºC	22ºC	32ºC
wt vs *phyA*	1.64	2.41	1.10
wt vs *phyB*	0.00	50.69***	12.93***
wt vs *phyD*	0.03	0.72	22.91***
wt vs *phyE*	0.34	0.45	0.11

Supplementary Table S2: Tests for effect of temperature on phytochrome transcript level over time. Results of Analysis of Variance of normalized gene expression of samples exposed to different temperatures collected at different time points during dark pre-incubation and after transfer to light. See Figure 1C for transcript levels over time. Sums of squares and F-values are given for each gene analyzed separately. df-Time = 3, df-Temperature = 1, df-Time x Temperature = 3. +P < 0.06; *P $\leq$ 0.05; ** P < 0.01; ***P < 0.001.

	PHYA		*PHYB*		*PHYD*		*PHYE*	
Factor	SS	F	SS	F	SS	F	SS	F
Time	46.33	27.25***	5.76	6.91**	10.40	20.41***	3.77	76.70***
Temperature	5.54	9.79**	0.247	0.89	0.07	0.40	0.01	0.51
Time x Temperature	5.20	3.06+	5.43	6.51**	16.07	31.52***	3.39	68.98***

Supplementary Table S3: Tests for effect of temperature on transcript level during and after high-temperature pre-incubation in the dark. Results of Analysis of Variance of normalized transcript level of samples exposed to different temperatures collected at different time points during dark pre-incubation and after transfer to light. See Figure 2A for transcript levels over time. Sums of squares and F-values are given for each gene analyzed separately. df-Time = 3, df-Temperature = 1, df-Time x Temperature = 3. +P < 0.10; *P ≤ 0.05; ** P < 0.01; ***P < 0.001.

Gene	SS-Time	F-Time	SS-Temp	F-Temp	SS-Time x Temp	F-Time x Temp
PHYD	7.13	2.54+	12.49	13.36**	16.19	5.77**
RD29a	24.05	40.59***	28.54	144.49***	12.15	20.51***
NCED6	61.48	93.21***	6.98	31.76***	18.68	28.32***
NCED9	60.69	20.18***	11.90	11.87**	12.72	4.23*
ABI5	17.09	16.95***	36.87	109.71***	2.70	2.68+
SOM	27.79	5.71**	20.31	12.52**	7.42	1.52
DOG1	8.04	324.47***	3.77	456.55***	4.51	182.09***
GA2ox2	122.54	55.42***	11.86	16.09**	25.17	11.38***
PIL5	7.48	3.10+	1.00	1.24	5.61	2.33
GA3ox1	37.45	15.52***	1.59	4.62*	25.52	10.58***
GA3ox2	27.62	4.63**	4.54	5.34*	30.73	5.16***

Supplementary Table S4: Tests for differences between L*er* wild-type and the *phyD* mutant in transcript level over time. Results of Analysis of Variance of normalized transcript level in L*er* wt and *phyD* mutant samples collected at different time points after dark pre-incubation and transfer to light. See Figure 3B for transcript levels over time. Sums of squares and F-values are given for each gene analyzed separately. df-Time = 3, df-Genotype = 1, df-Time x Temperature = 3. +P < 0.10; *P ≤ 0.05; ** P < 0.01; ***P < 0.001.

[a] The transcript level of *PHYD* did not change over time in the *phyD* mutant (F = 0.88, P > 0.05), but it did in the wt (F = 5.00, P = 0.01). The *phyD* mutant had lower levels of *PHYD* mRNA levels than wt in the dark (F = 23.74, P = 0.008) and at 1d after transfer to light (F = 7.43, P = 0.05), but no significant difference at the other time points.

Gene	SS-Time	F-Time	SS-Geno	F-Geno	SS-Time x Geno	F-Time x Geno
PHYD[a]	2.39	2.45+	0.32	1.69	2.10	2.16+
PIL5	3.64	10.18***	1.35	18.90***	0.54	1.52
RD29a	3.63	9.89***	0.64	8.77**	0.72	1.97
ABI5	7.44	31.99***	0.94	20.21***	0.70	3.01*
GA2ox2	63.43	8.92***	16.96	11.92**	34.96	4.91**

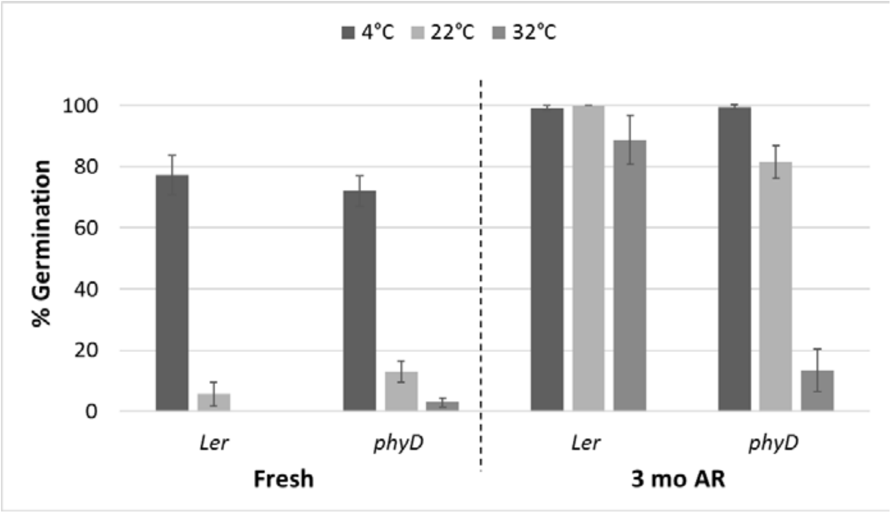

Figure S1. Primary dormancy in fresh and 3 months after-ripened seeds of L*er* wild-type and *phyD* mutant genotypes. Fresh seeds of the L*er* wt and *phyD* mutant, and seeds after-ripened for three months, were incubated on agar in the dark for 4 days at the indicated temperature (4°, 22°C or 32°C). After 4 days, seeds were transferred to 22°C,12h photoperiod, and radicle protrusion was monitored after 10 days. Error bars represent the standard error of 9 replicates of 15 seeds each.

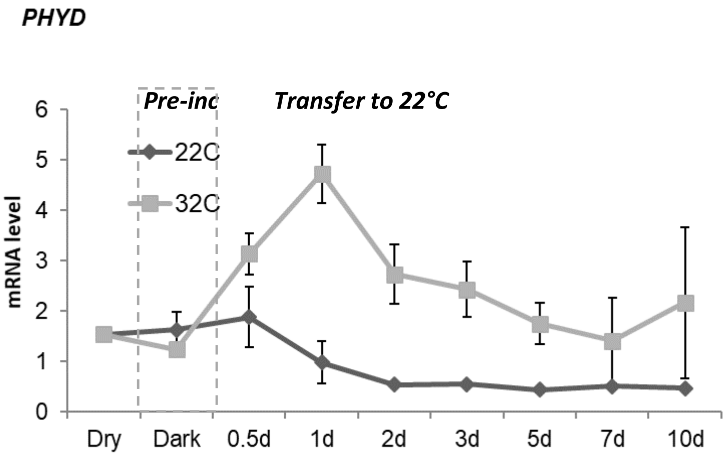

Figure S2. *PHYD* transcript level in L*er* wild-type seeds pre-incubated at two temperatures. Relative transcript level measured in dry seeds, 96h after pre-incubation in the dark at 22°C or 32°C (pre-inc) and following transfer to permissive germination conditions (22°C and 12h photoperiod) for 0.5 to 10days. Gene-specific transcript levels were normalized to the *AT4G34270* transcript (*TIP41-like*). Error bars indicate standard error of three biological replicates.

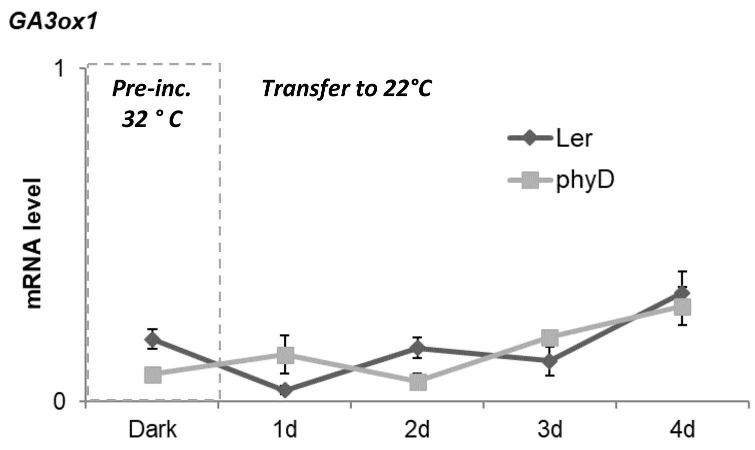

Figure 3. *GA3ox1* transcript level following high temperature pre-incubation. Transcript level in L*er* wild-type and *phyD* mutant seeds 96h after pre-incubation in the dark at 32°C (pre-inc) and 1d, 2d, 3d, and 4d after transfer to permissive germination conditions (22°C, 12h photoperiod). Transcript levels were normalized to the *AT4G34270* transcript (*TIP41-like*). Error bars indicate standard error of three biological replicates.
